# Comparative analysis of somatostatin analog uptake between successfully irradiated and non-irradiated meningiomas

**DOI:** 10.31744/einstein_journal/2022AO0104

**Published:** 2022-11-09

**Authors:** Guilherme de Carvalho Campos, Edson Amaro, Eduardo Weltman, Suzana Maria Fleury Malheiros, Bruna Letícia Ferrari, Taise Vitor, Marycel Rosa Felisa Figols de Barboza, Rosemeire Pereira Bezerra, Lilian Yuri Itaya Yamaga, Jairo Wagner, Ronaldo Hueb Baroni

**Affiliations:** 1 Hospital Israelita Albert Einstein São Paulo Brazil Hospital Israelita Albert Einstein, São Paulo, SP, Brazil.

**Keywords:** Meningioma, Receptors, somatostatin, Positron-emission tomography, Radiotherapy

## Abstract

**Objective:**

To evaluate whether there is a significant difference in somatostatin analog uptake in meningiomas treated or not with radiation therapy.

**Methods:**

A cross-sectional study was performed comparing measurements of somatostatin analog (^68^Ga-DOTATATE) uptake in two independent groups of ten patients each - one consisting of patients with meningiomas previously treated with radiation therapy and another comprising patients who had never been submitted to radiation therapy. All patients underwent PET/CT and MRI scans in an interval shorter than 24 hours between exams.

**Results:**

A total of 32 meningiomas from 20 patients were analyzed, all presenting significant somatostatin analog uptake in different degrees. The uptake levels of somatostatin analog were similar between the lesions treated or not with radiation therapy, and the mean values of SUV_max_ were 27.62 and 24.82, respectively (p=0.722). For SUV_mean_, the values were 16.20 and 14.82, respectively (p=0.822).

**Conclusion:**

Comparative analysis between the groups showed no significant differences in degree of somatostatin analog uptake in successfully irradiated and non-irradiated meningiomas.

## INTRODUCTION

Meningiomas are mesodermal lineage neoplasms that originate from arachnoid cap cells covering the brain and spinal canal and account for approximately 37% of central nervous system tumors.^(1)^ Although most meningiomas exhibit slow growth rate and benign behavior, in some patients these tumors may be associated with significant morbidity and mortality related to compression or direct invasion of adjacent structures, increased intracranial pressure, and changes in cerebrospinal fluid dynamics.^(2,3)^

According to the World Health Organization (WHO) classification, meningiomas are divided into three histological grades: benign (grade I), with lower risk of recurrence or aggressive behavior; atypical (grade II), and anaplastic or malignant (grade III).^(4)^ Recurrence rates reported in several series in the literature ranges from 7% to 25% for grade I meningiomas, 30% to 50% for grade II, and 50% to 94% for grade III.^(1,5–7)^

The therapeutic approach for patients with meningiomas requires a balance between effectiveness of different modalities for tumor control and possible side effects resulting from the proposed treatment. Asymptomatic patients with small lesions can be followed up based on observation and serial imaging studies.^(8–10)^ For symptomatic patients or those with evidence of lesion progression, treatment usually consists of surgery (gross total resection), surgery combined with radiation therapy, or radiation therapy alone (for high-risk surgical patients or tumors not safely accessible by surgery).^(10–17)^ Current radiation therapy techniques provide high rates of local control with clinical improvement or at least symptom stabilization in most patients with grade I or II meningiomas.^(16–18)^

Imaging plays a vital role in management of these patients. Contrast-enhanced computed tomography (CT), and magnetic resonance imaging (MRI) are routinely used for diagnosis (defining the limits of the lesion and its relation with contiguous tissues), as well as for treatment planning and follow-up.^(19)^ Moreover, molecular imaging methods, especially somatostatin receptor positron emission tomography (PET) imaging, have been recently proposed as adjuvant tools for meningioma evaluation.^(20)^ Several reports confirmed meningiomas overexpress somatostatin receptors, especially somatostatin receptor subtype 2 (SST2), regardless of their histological grade.^(21–29)^ Given its high sensitivity and excellent target-to-background contrast (due to very low uptake in normal brain tissue), somatostatin analogs PET imaging may add valuable information to structural modalities, particularly for lesion detection and delineation, radiation therapy planning, and post-treatment evaluation.^(20)^

Response assessment of meningiomas after radiation therapy can be particularly challenging because, even when a satisfactory clinical response to treatment is achieved, significant morphological changes of the lesion are often not characterized.^(17,30)^ Somatostatin receptor imaging could be interesting in this scenario since, in theory, if radiotherapy was effective in reducing the tumor cell population or promoting relevant metabolic changes in neoplastic tissue, this might modify the degree of somatostatin analog uptake by the lesion, which can be verified and quantified by PET imaging.

## OBJECTIVE

To evaluate whether there is a significant difference in somatostatin analog uptake in meningiomas treated or not with radiation therapy.

## METHODS

A cross-sectional study was conducted comparing gallium-68 (^68^Ga)-labeled dodecane tetraacetic acid-tyrosine-3-octreotate (DOTATATE) PET findings in two independent groups of patients diagnosed with cranial meningiomas. Ten patients older than 18 years were recruited for each group. Group 1 comprised patients submitted to radiotherapy (as isolated treatment or complementary modality for residual lesion after surgery). According to the inclusion criteria, all patients in this group had completed radiation therapy more than one year before the study, and the treatment was considered successful (patients presented clinical improvement or at least clinical stability after treatment, and showed no signs of lesion progression in serial imaging studies). Group 2 included patients who had not undergone radiotherapy for meningioma. Patients with previous surgical treatment were included in both groups, provided they had residual lesion detectable in MRI. For patients who did not undergo surgery, the diagnosis of meningioma presumed by imaging techniques was accepted. For this study, patients underwent ^68^Ga-DOTATATE PET/CT and MRI imaging on the same day. The study protocol was approved by the local ethical committee (Hospital *Israelita Albert Einstein*; CAAE: 38602514.2.0000.0071; # 888.025) and was carried out in accordance with The Code of Ethics of the World Medical Association (Declaration of Helsinki) for experiments involving humans. All patients gave informed consent.

### ^68^Ga-DOTATATE synthesis

Gallium-68 was obtained from an IGG 100 ^68^Ge/^68^Ga-generator (Eckert and Ziegler, Berlin, Germany) and synthesis of [^68^Ga]Ga-DOTATATE was conducted in the automated Modular-Lab PharmTracer module (Eckert and Ziegler, Berlin, Germany) under good manufacturing practice (GMP) standards for in-house production, at a hospital radiopharmacy. DOTATATE acetate (GMP) precursor and the synthesis cassettes were acquired from ABX Advanced Biochemical Compounds (Radeberg, Germany). Briefly, [^68^Ga]GaCl_3_ is purified in an anionic resin to discard metal impurities and ^68^Ge residues, and added to reaction vials containing DOTATATE 40μg/2mL 0,1M acetate buffer. After 5 minutes of reaction time at 85^o^C, the product was purified by a Sep-Pak C_18_ cartridge. The final product was sterilized by Millipore 0.22µm filter. Radiochemical yield was determined by measuring the activity retained in the module and its proportion to the final product. Radiochemical purity was determined by Sep-Pak C_18_ and instant thin layer chromatography-silica-gel (ITLC-SG) strip using 1M ammonium acetate and methanol (1:1) and confirmed by high performance liquid chromatography (HPLC). Radionuclide identity was measured by decay analysis of ^68^Ga half-life using an ionization chamber. The pH value, microbiological purity, and pyrogenic test were done in each batch. The radiopharmacy team responsible for the labeling procedure has specific training and extensive experience for in-house production of ^68^Ga-labeled radiopharmaceuticals (^68^Ga-DOTATATE, as well as ^68^Ga-PSMA-11).^(31,32)^

### ^68^Ga-DOTATATE PET/CT imaging

PET/CT imaging was performed 50 minutes after intravenous radiopharmaceutical injection (74MBq) on a Biograph mCT scanner (Siemens Medical Solutions, Germany). PET data were acquired for 12 minutes and reconstructed with an iterative algorithm (8 iterations; 21 subsets) in a 400x400 matrix. Scatter correction and time-of-fly data were incorporated into the reconstruction process and CT was used for attenuation correction.

### Magnetic resonance image

Magnetic resonance image studies were performed on a 3.0T scanner (Tim Trio, Siemens Medical Solutions, Germany). T1-weighted images (before and after paramagnetic contrast administration), and T2-weighted sequences were acquired in multiples planes.

### Imaging analysis

Visual analysis of both imaging modalities was conducted by a radiologist and a nuclear medicine physician in cooperation, both board-certified and with expertise in neuro-oncology. After identification of the lesions, PET standard uptake values (SUV_max_ and SUV_mean_) were determined and metabolic tumor volume (MTV) was calculated using an automatic threshold-based method on PET images (threshold was set to 40% of SUV_max_ to delineate the lesions).

### Statistical analysis

The demographic data of both groups were described as absolute and relative frequencies. Quantitative variables were described by means, medians and 95% confidence intervals (95%CI) and analyzed by boxplot graphics. The Shapiro-Wilk test was used to verify the adequacy of quantitative data to normality and Student’s *t*-test and Mann-Whitney hypothesis tests were used for comparative analysis between the groups. Finally, the measures of SUV_max_, SUV_mean_, and MTV were compared using generalized linear mixed models, with gamma family and log link function (considering the dependency factor resulting from the occurrence of more than one lesion in some of the studied patients).

The statistical software package SPSS version 20 was used for descriptive analysis and graphics construction. For the generalized linear mixed models, the statistical packages R and Ime4 were used. Significance was defined as p<0.05.

## RESULTS

A total of 32 meningiomas were identified and analyzed in 20 patients enrolled in the study. Demographics of the two groups of patients are summarized in table 1, as well as information regarding previous treatments, number of lesions per patient, and histological classification.

**Table 1:** Demographics of 20 patients studied

Variables analyzed		Previous radiation therapy		p value
		Yes (n=10)	No (n=10)	
Female sex, n (%)		9 (90)	9 (90)	
Age (years)				
	Mean	52.40	58.80	0.224
	95%CI	47.08-57.72	48.61-68.99	
	Median	52.50	62.00	
Body mass index				
	Mean	24.38	27.85	0.075
	95%CI	22.14-26.61	24.60-31.10	
	Median	25.05	26.75	
Previous surgical treatment, n (%)				
	No previous surgery	3 (30)	6 (60)	
	One surgery	4 (40)	4 (40)	
	Two surgeries	3 (30)	0	
Number of lesions, n (%)				
	Single lesion	7 (70)	6 (60)	
	Two lesions	2 (20)	2 (20)	
	More than two lesions, n (%)	1 (10)	2 (20)	
WHO histological classification, n (%)				
	Grade I	4 (40)	2 (20)	
	Grade II	3 (30)	2 (20)	
	Grade III	0	0	
Radiologically presumptive diagnosis*, n (%)		3 (30)	6 (60)	

* patients who did not undergo surgical treatment.

95%CI: 95%confidence interval; WHO: World Health Organization.

Although unpaired, both groups consisted of nine women and one man. There was no significant difference in mean age or body mass index of patients between both groups.

From the group of patients previously treated with radiotherapy, 7 (70%) underwent radiation therapy as a complementary modality to surgery to treat a residual lesion. In the remaining three patients of this group, radiation therapy was the only treatment performed. From the group of patients who did not undergo radiotherapy, 4 (40%) had been previously treated with surgery. In the remaining (60%), no specific treatment for meningioma had been prescribed at the time of the study (some of these patients were scheduled for surgery or radiation therapy).

Regarding the WHO histological classification, six patients had diagnosis of grade I meningioma (four patients from the group previously treated with radiation therapy and two from the group with no previous radiotherapy), and five patients had diagnosis of grade II meningioma (three from the group previously treated with radiation therapy and two from the group with no previous radiotherapy). For the remaining nine patients, diagnosis of meningioma was presumed by imaging exams and therefore histological classification was not available.

### Radiation therapy

All patients previously treated with radiation therapy had received treatment at our organization, with doses ranging from 50.4Gy to 54Gy (28 to 30 sessions of 1.8Gy). In four patients (40%), the methodology employed was fractional stereotactic radiation therapy. In the remaining patients (60%), the volumetric modulated arc therapy methodology was used. Seven of 10 patients previously treated with radiotherapy had symptoms related to meningioma before treatment. After radiation therapy, four of these patients (57%) reported symptom relief (the remaining three patients reported stable clinical condition after treatment).

### Clinical and serial imaging follow-up

For the group of patients previously treated with radiation therapy, the clinical follow-up was defined as the period between the end of radiotherapy and the date of imaging studies. The mean follow-up time in this group was 7.2±4.3 years (mean ± standard deviation), with a minimum follow-up of 16 months. Clinical information and imaging exams of this period were reviewed, confirming there was no progression of the lesion after radiotherapy. The long follow-up period of these patients is justified because we intentionally selected patients for whom radiation therapy was effective, with consolidated results (since meningiomas usually grow slowly, the selection of patients with extended observation periods after radiotherapy is essential to study the effects of this treatment). For the group of patients with no previous radiotherapy, the follow-up period (defined as the time from diagnosis to imaging exams) was significantly shorter (mean follow-up of 1.8±1.7 years).

### Somatostatin analog uptake in meningiomas

**Figure 1:**
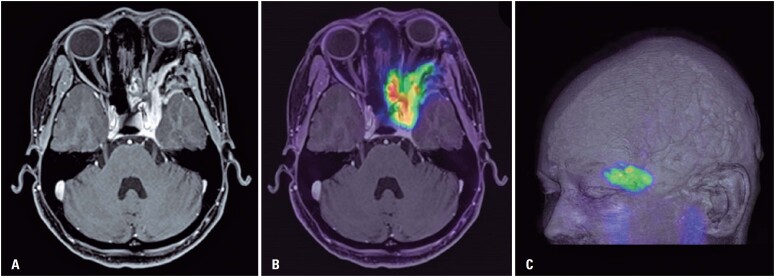
Example of a skull base meningioma with high somatostatin analog uptake. The tumor extends to the cavernous sinus and the left orbital and nasal cavities. Axial magnetic resonance image contrast enhanced T1-weighted sequence (A); PET/MRI fusion image (B) and 3D volume rendering (C)

**Figure 2:**
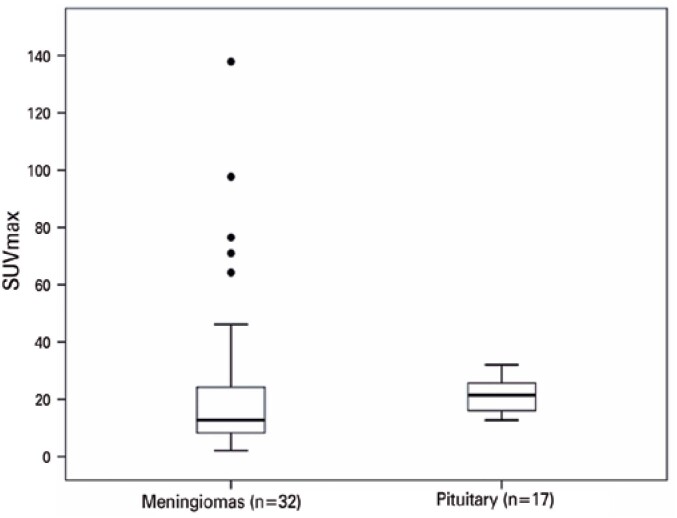
Boxplot for SUV_max_ in 32 meningiomas and normal pituitary tissue

All meningiomas showed significant somatostatin analog uptake, in different degrees, with SUV_max_ ranging from 2.15 to 137.92. Figure 1 shows an example of a skull base meningioma with high radiopharmaceutical uptake (SUV_max_: 20.2) and table 2 shows the central tendency and dispersion measures for somatostatin analog uptake variables (SUV_max_ and SUV_mean_) in meningiomas. The same parameters were calculated for the pituitary gland, used as a reference tissue with physiological somatostatin analog uptake (pituitary measurements were performed in 17 patients only, since the other three presented pituitary fossa invasion by the tumor, making it impossible to properly delimit the normal pituitary tissue). Figure 2 shows a boxplot for SUV_max_ in 32 meningiomas and also in normal pituitary tissue. There is a greater dispersion of measurements in the meningiomas compared to the pituitary gland (evidenced by wider interquartile range (IQR) and greater distance between the minimum and maximum values, as well as some discrepant measurements in the lesions).

**Table 2:** Central tendency and dispersion measures for somatostatin analog uptake variables in meningiomas and normal pituitary tissue

	Meningiomas (n=32)		Pituitary gland (n=17)	
	SUV_max_	SUV_mean_	SUV_max_	SUV_mean_
Mean	25.70	15.25	21.97	13.04
Median	12.72	7.03	21.55	12.89
Minimum	2.15	1.20	12.75	8.21
Maximum	137.92	86.13	32.03	18.70
IQR	17.05	9.47	9.33	5.23

IQR: interquartile range.

### Comparative analysis

**Figure 3:**
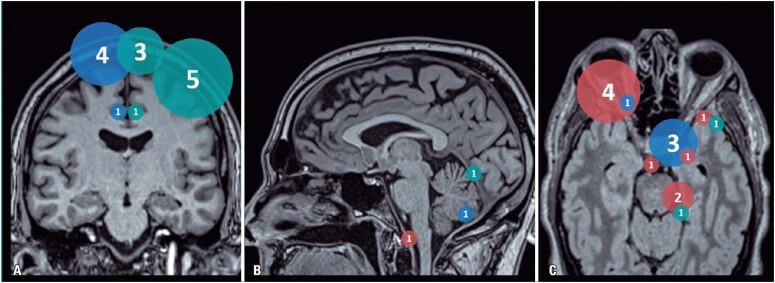
Location of the analyzed meningiomas. A normal MRI study in the coronal (A), sagittal (B) and axial (C) planes was used as a template

For comparative analysis, the meningiomas were divided into three clusters: a) irradiated main lesions; b) non-irradiated main lesions; and c) secondary lesions. The main lesions corresponded to the index lesions, which motivated the treatment or medical follow-up of patients. Secondary lesions corresponded to other meningiomas detected in imaging studies during the evaluation. Secondary lesions were significantly smaller compared to the main lesions and were more frequently located in the cerebral convexity (the main lesions were located mostly at skull base). This difference in size and location could be related to differences in biological and metabolic behavior, therefore we decided to analyze secondary lesions separately (Figure 3 shows the location of the analyzed meningiomas).

**Figure 4:**
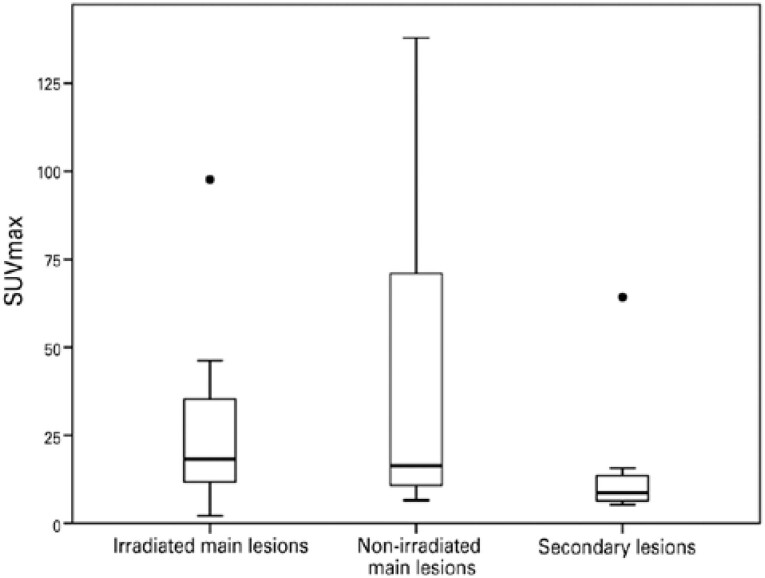
Boxplot for SUV_max_ of irradiated and non-irradiated main lesions and secondary lesions

Table 3 shows the uptake and metabolic volume measurements of the irradiated and non-irradiated meningiomas. Generalized linear mixed models showed no significant differences in somatostatin analog uptake levels between the irradiated and non-irradiated lesions. Figure 4 shows a boxplot for SUV_max_ of main lesions (irradiated and non-irradiated) and secondary lesions.

**Table 3:** Uptake and metabolic volume measurements of the irradiated and non-irradiated meningiomas

Measurements		Irradiated lesions (n=10)	Non-irradiated lesions* (n=22)	Mean ratio (95%CI)	p value
SUV_max_				1.12 (0.60; 2.10)	0.722
	Mean (SD)	27.62 (27.74)	24.82 (33.20)		
	Median (IQR)	18.25 [12.44; 32.13]	10.98 [7.25; 19.15]		
	Min-Max.	2.15-97.70	5.26-137.92		
SUV_mean_				1.08 (0.57; 2.03)	0.822
	Mean (SD)	16.20 (17.50)	14.82 (20.83)		
	Median (IQR)	10.08 [7.26; 18.34]	5.90 [3.96; 10.79]		
	Min-Max.	1.20-61.81	3.03-86.13		
MTV 40% (cm^3^)				3.34 (1.56; 7.11)	0.002
	Mean (SD)	4.02 (3.89)	1.90 (2.45)		
	Median (IQR)	3.28 [1.13; 5.35]	0.97 [0.35; 1.33]		
	Min-Max.	0.28-13.21	0.13-8.09		

* Non-irradiated meningiomas include primary and secondary lesions.

SD: standard deviation; IQR: interquartile range; Min-Max: minimum and maximum values; 95%CI: 95% confidence interval; MTV: metabolic tumor volume.

Metabolic tumor volume, however, is significantly different between groups, with lower values for secondary lesions (but similar between main lesions treated or not with radiotherapy).

**Figure 5:**
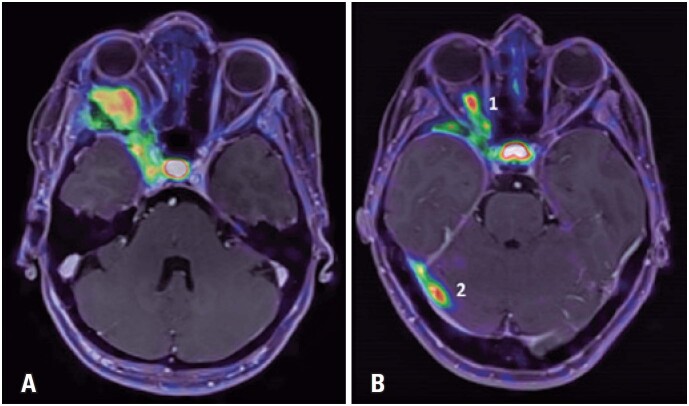
PET/MRI fusion images of two patients. The first patient (A) had a meningioma treated with surgery plus radiotherapy, representing an irradiated main lesion (SUV_max_: 9.55 and SUV_mean_: 4.85). The second patient (B) had a meningioma involving the optic nerve (1) treated with surgery alone, representing a non-irradiated main lesion (SUV_max_: 10.8 and SUV_mean_: 5.62) and a secondary lesion (2) in the tentorium (SUV_max_: 8.63 and SUV_mean_: 5.22). Somatostatin analog uptake was similar in the three lesions and physiological uptake in the pituitary gland was evident in both patients

Figure 5 shows an example of PET/MRI fusion images of two patients. The first patient (A) had a meningioma treated with surgery plus radiotherapy (representing an irradiated main lesion). The second patient (B) had a meningioma involving the optic nerve treated with surgery alone (representing a non-irradiated main lesion) and a secondary lesion in the tentorium (this patient was being evaluated for complementary radiotherapy of the main lesion). Somatostatin analog uptake is similar between the three lesions. Physiological uptake in the pituitary gland is evident in both patients.

## DISCUSSION

Contrast-enhanced CT and especially MRI are the main methods for diagnosis and follow-up of meningiomas, providing high spatial resolution images and accurate delineation of neoplastic tissue. Nevertheless, since these methods are based primarily on anatomical features, in some specific situations the information provided may be insufficient, especially when structural changes are not the main aspect to be analyzed.^(20)^ In this context, evaluation of radiation therapy response can be particularly challenging because, even when the treatment has been considered successful, images often persist without significant changes in follow-up studies. A methodology that allows earlier and more assertive assessment of effects of radiotherapy on meningiomas would be of major clinical importance. Early identification of patients most likely to relapse is important for prognostic definition and indication of complementary treatments. On the other hand, stating the treatment was effective may reduce the need for multiple serial exams and long-term follow-up. The present study aimed to evaluate whether somatostatin receptor PET images can assist in this analysis, providing evidence of the effects of radiation therapy on meningiomas.

### Somatostatin receptor density in meningiomas

Before the comparative analysis between the groups, a first noteworthy finding is the pronounced variability in degree of somatostatin analog uptake observed in meningiomas. Both uptake variables evaluated (SUV_max_ and SUV_mean_) presented a wide range of results. The SUV_max_, e.g., ranged from 2.15 to 137.92 in meningiomas, with an IQR of 17.05. In comparison, normal pituitary tissue, used as a reference for physiological uptake, presented SUV_max_ ranging from 12.75 to 32.03, with an IQR of 9.33. This finding indicates a pronounced heterogeneity of somatostatin receptor density in meningiomas, which certainly influences the evaluation of these tumors using this methodology; therefore, it should be considered in the interpretation of results.

### Comparative analysis between the groups of irradiated and non-irradiated lesions

Comparative analysis, the main objective of this study, showed no significant difference in somatostatin analog uptake in meningiomas treated or not with radiation therapy (considering both investigated variables, SUV_max_ and SUV_mean_).

Despite its wide use in the treatment of meningiomas, the mechanisms of action of radiotherapy (in its different modalities) to control these lesions have not been completely understood. In addition to direct cell toxicity, effects on cell replication (resulting from DNA damage) and apoptosis induction have been suggested.^(33)^ However, considering the relatively low degree of cell replication usually observed in grade I and II meningiomas, additional mechanisms are likely to act in combination. Vascular changes in the neoplastic tissue as well as in the tumor microenvironment probably represent a pivotal effect.^(34)^ Obliteration of arterioles and capillaries resulting from radiation-induced endothelial dysfunction, a well-known effect in other neoplasms, could cause a global reduction in cell metabolic activity, thereby inhibiting cell replication.^(33)^

Based on assumptions and concepts similar to those employed in this study, Gudjonsson et al.^(33)^ published an article on evaluation of 19 meningioma patients before and after proton therapy. The authors reported an average reduction by 19.4% in methionine uptake by meningiomas after radiotherapy, although no significant reduction in lesion dimensions was observed, suggesting radiolabeled amino acid PET images may early depict the effects of proton treatment in these patients. In accordance with these results, lower levels of somatostatin analog uptake could be expected in meningiomas effectively treated with external beam radiation therapy as compared to non-irradiated lesions (either by a reduction in the neoplastic cell population or by changes in the tumor microenvironment), but this was not observed in our study. Nonetheless, the substantial methodological differences employed in the studies should be noted. First, Gudjonsson et al. evaluated patients undergoing proton therapy and the tracer used was labeled methionine (MET-^11^C); in other words, the amino acid metabolism was analyzed.^(33)^ In addition, the radiopharmaceutical uptake level was longitudinally compared (before and after treatment) in each lesion. On the other hand, in the present study, meningiomas of two independent groups of patients were compared, and the tracer used was a somatostatin analog, thus assessing the density of somatostatin receptors in the lesions.

The substantial uptake of the somatostatin analog in radiotherapy-treated meningiomas (at similar levels to those observed in untreated lesions) indicates a significant number of viable and metabolically active neoplastic cells persist in these tumors (since somatostatin receptor expression depends on cellular activity).^(35,36)^ This statement is not intended to question the effectiveness of radiation therapy for treatment of meningiomas. On the contrary, as mentioned in the methodology section, to compose the group of treated lesions, patients in whom radiotherapy was considered successful were intentionally selected. A basic principle of ionizing radiation treatment is that tumor cells, usually showing a higher rate of replication, are more sensitive to DNA radiation damage than normal tissues.^(37)^ However, as stated above, it is currently accepted that other mechanisms are involved in tumor control by radiation therapy, especially in neoplasms with low rates of cell replication. These mechanisms include loss of induction of cell cycle pause, senescence, apoptosis, as well as effects on the tumor microenvironment and vascular supply.^(33,34,38)^ Such effects may occur in meningiomas undergoing radiation therapy, not necessarily affecting somatostatin receptor expression in a significant way.

Another approach for the use of somatostatin analog PET studies in meningiomas was explored by Sommerauer et al.^(39)^ These authors evaluated somatostatin analog uptake in a series of 45 patients with meningiomas. Their purpose was not to analyze treatment effects but to correlate the degree of radiopharmaceutical uptake with tumor growth rate. The results indicated a strong correlation between SUV_max_ values and tumor growth rate for grade I and II intracranial meningiomas, suggesting somatostatin receptor PET studies could be used to predict patients at higher risk for progression.^(39)^ Although the studies have markedly different purposes, it is interesting to analyze the results in parallel. The cluster of irradiated lesions was carefully selected for our study and showed no clinical or imaging signs of progression after treatment (with a long follow-up period of 7.2±4.3 years). Nevertheless, these meningiomas still showed significant uptake of the somatostatin analog (at levels similar to those observed in non-irradiated lesions). The wide variability of the degree of somatostatin analog uptake observed in meningiomas, even in irradiated lesions, suggested that despite the correlation pointed by Sommerauer et al.,^(39)^ it may be difficult to establish a cut-off value to discriminate between lesions that will or will not progress (based only on the uptake value).

No other studies specifically investigating the use of somatostatin analogs to depict the effects of radiotherapy on meningiomas were found in the literature.

### Study limitations

This is a proof-of-concept study with a limited number of patients designed to explore the potential of somatostatin receptor imaging for post-radiotherapy meningioma evaluations. Certainly, a study with a larger number of evaluated lesions could bring stronger results. The study design, a cross-sectional analysis of two independent groups of patients, is another topic that can be considered a limitation. A prospective study comparing the same patient before and after treatment would be a more direct option to answer whether radiation therapy promoted any significant change in radiopharmaceutical uptake in meningiomas. A prospective study, however, would require more time, especially to evaluate the late effects of treatment. The option for a cross-sectional comparison between two independent groups, therefore, allowed the selection of patients with long follow-up periods and effective treatment already established.

## CONCLUSION

Imaging plays a vital role in the management of meningiomas, and somatostatin receptor PET studies have been recently proposed as an adjuvant tool for evaluation of these tumors, particularly for lesion detection and delineation, radiation therapy planning, and post-treatment evaluation. But there are few publications specifically evaluating the use of molecular imaging to assess the effects of radiotherapy on meningiomas. To the best of our knowledge, this is the first study addressing this issue with somatostatin analogs.

Due to the aforementioned limitations, the study does not allow us to conclude that radiation therapy has no effect on somatostatin receptor density in meningiomas. However, our study clearly showed a pronounced variability in the degree of somatostatin analog uptake in meningiomas, and significant levels of uptake in successfully irradiated lesions (sometimes at levels similar to those observed in untreated lesions). Hence, somatostatin analog uptake in meningiomas after radiation therapy should not be interpreted as treatment failure.
